# The mediating role of intention of learning behaviour in learning behaviour

**DOI:** 10.3389/fpsyg.2024.1228783

**Published:** 2024-02-26

**Authors:** Xianhui Li, Zhanjun Wang, Jingyu Xie

**Affiliations:** College of Teacher Education, Zhejiang Normal University, Jinhua, China

**Keywords:** learning attitude, learning motivation, self-efficacy, the intention of learning behaviour, learning outcome

## Abstract

Improving the quality of postgraduate study is one that must be addressed with the increase in the number of postgraduate students. This study aims to analyse the effects of learning attitude, learning motivation and self-efficacy on learning behaviour through the intention of learning behaviour, and the effect of learning behaviour on learning outcome. Measurements were made on 560 postgraduate students after the development of a scale. The scale was analysed for reliability and exploratory factor analysis using SPSS software. The date were then analysed using structural equation modelling (SEM) analysis techniques with path analysis and bootstrap methods. The results of the study showed that students’ attitudes towards learning and self-efficacy had a significant indirect on learning behaviour through the mediating involvement of intention to learn behaviours, and learning behaviour had a significant effect on learning outcome. Therefore, there is a need to improve learning behaviour by improving students’ intention to learn behaviour so that they can have good learning outcome.

## 1 Introduction

In recent years, the scale of postgraduate education has increased significantly with expanding postgraduate enrolments, challenging the quality of postgraduate training, of which students’ learning behaviours are an important response. Learning behaviour is an essential indicator of student learning effectiveness and has long occupied an important position in studying student learning (Sedrakyan et al., 2020; Hwang et al., 2021). Domestic and international scholars consider learning behaviour a key factor in determining students’ learning effectiveness (Anthony, 2000; David and Mohamad, 2006). The famous behaviourists, such as Skinner, Bandura, and Thorndike, attributed great importance to learning behaviour, which is considered a fundamental dimension of learning. Furthermore, there is a significant positive relationship between student learning behaviours and learning outcomes; that is, improving student learning behaviours can improve learning outcome (LO) to a certain extent (Durbrow et al., 2000; Valaitis et al., 2005; Carini et al., 2006; Keengwe and Bhargava, 2014; Huang, 2022). And the LO including a variety of competency gains and value gains (Van Uum and Pepin, 2022). So learning behaviour is undoubtedly an important response to the quality of postgraduate training from an individual development perspective.

Learning behaviour (LB) is including learning in class, extracurricular learning practice in and assignment completion (Hsieh and Hsieh, 2019; Jovanović et al., 2021). So learning behaviour can be observed through the responses given by students to learning situations interacting with academic assignments (Mcdermott et al., 2012; Chao et al., 2018). Literature studies in previous studies reveal that many factors influencing student learning behaviour, these factors can be seen in more detail in three main groupings: (1) learning attitudes (Rahman et al., 2012; Rikoon et al., 2012); (2) self-efficacy (Bandura and Schunk, 1981; Locke, 1997; Elliot, 1999; Zimmerman, 2000; Elliot and McGregor, 2001; Geitz et al., 2016); (3) learning motivation (Tokan and Imakulata, 2019; Pawlak et al., 2020; Singh et al., 2022).

Learning attitude (LA) is an internalised, dispositional psychological phenomenon that plays an important role in our behaviour and subjective feelings (Deqing, 2001). Between attitude and behaviour, there exists an internal dispositional attitude towards a particular object that transforms an individual’s subjective perception into concrete behaviour in real life (Deqing, 2001). The results of one study indicate the direct effect of attitudes on behaviour is weak, so the researcher suggested a future research to test the learning culture as a moderator variable that can provide strengthening of behaviour (Sondeng et al., 2020). Thus, the influence of learning attitudes on learning behaviour may be indirect.

Learning motivation (LM) including both intrinsic motivation(IM; what a person inherently wants to do because of internal stimuli) and extrinsic motivation (EM; a response to an external stimulus like praise, rewards, or punishment; Harter, 1981; Linnenbrink and Pintrich, 2002; Singh et al., 2022), which also has an impact on LB (Bosch et al., 2021). Intrinsic motivation is defined as motivation to engage in an activity for its own sake, whereas extrinsic motivation refers to motivation to engage in an activity as a means to an end (Sichler, 2014). There are now well-established scales for assessing intrinsic and extrinsic motivation for students at all stages of learning, such as the Harter (1981) scale and Motivated Strategies for Learning Questionnaire (MSLQ; Pintrich et al., 1993). The findings of most researchers indicate that motivation has a direct impact on learning behaviour (Tokan and Imakulata, 2019; Singh et al., 2022). But whether there is a mediating variable between the two is unknown.

Self-efficacy (SE) has been defined as individuals’ beliefs about their performance capabilities in a particular context or a specific task or domain (Locke, 1997). Students who have more positive self-efficacy beliefs are more likely to work harder, persist, and eventually achieve at higher levels (Linnenbrink and Pintrich, 2002; Jaedun et al., 2022). There is evidence that self-efficacy can positively influence students’ learning behaviour and will challenge them with difficult tasks, such as choosing a higher mathematics course at school (Eccles et al., 1998; Kholifah et al., 2023). Self-efficacy beliefs have been found to be sensitive to subtle changes in students’ performance context, to interact with self-regulated learning processes, and to mediate students’ academic achievement (Zimmerman, 2000). Therefore, self-efficacy has an indirect effect on learning behaviour.

Intentions have been defined in the TPB as: the amount of effort one is willing to exert to attain a goal, and the stronger the intention to engage in a behaviour, the more likely it is that the goal of the behaviour will be achieved (Ajzen, 1991). As a general rule, these intentions account for considerable variance in actual behaviour (Ajzen, 1991). In essence, intentions can be conceived of as goal states in the expectancy value tradition that are the result of a conscious process that takes time, requires some deliberation, and focuses on consequences (Loewenstein et al., 2001). And the intentions of learning behaviour (ILB) is the most proximal predictors of actual learning behaviour (Gatch and Kendzierski, 1990; Froehlich et al., 2023). Therefore, intention to learn behaviour is an important mediating variable.

In this context, based on the concepts and the theory of planned behaviour (Dzewaltowski et al., 1990; Ajzen, 1991), the postgraduate students’ behaviour is interesting to be researched. This study focuses on measuring the significance of LA, LM and SE on the LB, and measuring the role of ILB in mediating LA, LM and SE influencing LB. Furthermore, exploring the impact of LB on learning outcome. Thus, the hypothesis of the study: (1) LA has a positive influence on ILB; (2) LM has a positive influence on the ILB; (3) SE has a positive influence on ILB; (4) IBL has a positive influence on LB; (5) LB has a positive influence on LO; (6) There is a significant positive effect of LA on the LB mediated by ILB; (7) There is a significant positive effect of LM on the LB mediated by IBL; (8) There is a significant positive effect of SE on the LB mediated by IBL.

## 2 Research method

### 2.1 Research design

Some researchers have studied the meaning and influencing factors of learning behaviour (McDermott et al., 2002; Yen et al., 2004; Escalón and Greenfield, 2009; Klug et al., 2013). Research has been done on different age groups and scales of learning behaviour have been developed (Spivack and Swift, 1966; Reynolds, 1979; Mcdermott, 1999), such as the Learning behaviours Scale (LBS) was developed in the United States. This study based on the theory of planned behaviour, emphasising the indirect influence of LA, LM, and SE on LB and the results of the structural analysis based on the IBL as a mediator.

### 2.2 Study sample

From October 2022 to March 2023, a combination of random sampling and purposive sampling was used to select 10 universities in China. Of these, purposive sampling was used to obtain representative proportions for the local universities and random sampling was used for the external universities. The study population was enrolled master’s students. A total of 560 copies of the scale were randomly distributed and 560 copies were collected, with a recovery rate of 100%. After the check and selection of scale, three invalid scales were excluded and 557 scales were valid, with an effective rate of 99.5%. Among them, 20.8% were male and 79.2% were female; 65.9% were between 20 and 25 years old, 30.5% were between 26 and 30 years old and 3.6% were over 30 years old; and 32.9% majored in science and technology, and 67.1% majored in literature and history. The proportion of full-time master’s degree students is 74%, part-time master’s degree students is 3.6%, full-time professional master’s degree is 16.2%, and part-time professional master’s degree is 6.3%; The proportion of first-year graduate students is 51.3%, second-year graduate students is 29.1%, and third-year graduate students is 19.6%.

### 2.3 Date collections

The date in the research on LA, LM, SE, ILB, LB, and LO variables were collected using online-based questionnaire technique. The overall scale consists of four parts: basic information, factors that influence learning behaviour, learning behaviour, and learning outcome. Except for basic information, all questions were compiled using a five Likert scale. And different variables have different answer options. LA, LM, SE, and ILB consisting of answer options not at all, not matching, general compliant, fully compliant. LB consisting of answer options never, sometime, normally, usually, always. LO consisting of answer options not improved, a little improved, general, substantially improved, great improved. In order to ensure the reliability and validity of the scale, a small-scale pilot test of the Master’s Learning Behaviour Influence Factor Scale was conducted before the official test. The preparation of the scale was carried out concerning the study of literature and expert opinion. The specifics of scale are shown in Table 1.

**Table 1:** Summary of measurement instrument, construct loadings, reliabilities, and references.

Construct		Measurement items	Loading	Cronbach’s α	References
FILB	LA	I’ll spend most of my time outside of class studying and researching.	0.771	0.940	National survey of student engagement (NSSE, 2019) The Learning Behaviours Scale (LBS; Mcdermott, 1999) Student Learning: Attitudes, Engagement and Strategies (OECD, 2004)
		Study all the courses carefully.	0.735		
		I’ll develop study and research plans and endeavour to put them into action.	0.614		
		I’ll carefully read and write about the books recommended by the tutor.	0.567		
	LM	I’ll study hard for the scholarship.	0.716		
		Other people’s success stories are my motivation to learn.	0.715		
		I’ll study hard to improve my social status in the future.	0.684		
		My teacher’s strict requirements are my motivation to keep learning.	0.624		
		I’ll study hard to find a job in the future.	0.615		
		In order to be able to graduate, I conducted serious academic research.	0.592		
		In order to have a competitive edge in the future, I will study different fields of specialisation.	0.528		
	SE	If I put in the effort, I’ll be able to solve my learning difficulties	0.793		
		When there’s a learning difficulty, I believe it’s always solved.	0.706		
		I’m sure I’ll pass my semester exams with flying colours.	0.654		
	ILB	I like to use concept maps or mind maps to summarise knowledge.	0.778		
		I can always make connections between new knowledge and old knowledge I’ve already learnt.	0.721		
		I like to keep thinking and reviewing important knowledge until I fully understand it.	0.694		
		I’m happy to spend time on academic research.	0.683		
		I’m happy to learn new things.	0.635		
		I like the courses in my major.	0.493		
LB	LBC	Active participation in questions or discussions in class.	0.830	0.919	
		Presenting and arguing opinions or ideas in class.	0.790		
		Communicate with the teacher when you have questions in class.	0.741		
		Prepared classroom presentations on a particular topic.	0.739		
	CA	Participation in teachers’ projects.	0.791		
		Participation in various academic conferences and seminars.	0.781		
		Writing learning summaries.	0.774		
	ELP	Reading various books.	0.762		
		Use of holidays for learning.	0.734		
		Discussing assignments with classmates during class.	0.719		
		Use of electronic media for discussion and completion of assignments (e.g., online classes, web forums, chat tools, et al.).	0.677		
		Discuss with classmates after school about what they are studying or problems they are having.	0.551		
LO	CG	Oral expression	0.820	0.953	
		Expertise and skills	0.780		
		Written capacity	0.764		
		Organisational leadership skills	0.763		
		Ability to use information technology	0.738		
		Critical thought	0.721		
		Wide range of areas of knowledge	0.698		
		Solving complex problems in reality.	0.688		
		Working effectively with others.	0.677		
	VG	Establishing outlook on life and values.	0.842		
		Self-recognition	0.810		
		Define your future development plans	0.789		
		Understanding the culture and values of different groups.	0.758		

FILB, factors of influencing learning behaviour; LA, learning attitude; LM, learning motivation; SE, self-efficacy; ILB, intention of learning behaviour; LB, learning behaviour; LBC, learning behaviour in class; CA, completion of assignments; ELP, extracurricular learning practice; LO, learning outcome; CG, capability gains; VG, value gains.

### 2.4 Date analysis

Structural Equation Modelling (SEM) analysis was used to test the hypothesis on the effect of exogenous variables on endogenous variables. Testing the validity and reliability of the instrument using confirmatory factor analysis (CFA). Path analysis was used to measure the effects produced between the variables. Also, the bootstrap method was used to measure the mediating role of learning behavioural intentions. In this study, LA, LM and SE were used as exogenous variables, LB as endogenous variables and IBL as an intervening variable. Amos 24 software was used in this study. The research hypotheses are based on the support of theories related to exogenous variables to endogenous variables, as presented in the literature review above.

## 3 Findings

### 3.1 Reliability analysis

Homogeneity reliability and split-half reliability were used to analyse the reliability of the scales. Among them, the reliability of homogeneity was expressed by the internal consistency coefficient (Cronbach’s α). The results showed that Cronbach’s α coefficient and the split-half reliability of each index reached greater than 0.8; thus, the reliability was good. The details are shown in Table 1.

### 3.2 Exploratory factor analysis

The KMO and Bartlett’s spherical test were used to perform exploratory factor analysis on all scales, and adjusted and censored for the items by SPSS24 software. The results showed that the KMO values of each component of the influencing factor of learning behaviour, learning behaviour, learning outcome were 0.940, 0.918, and 0.952, respectively. The k chi-square values of Bartlett’s spherical test were 5867.813, 3407.629, and 5565.727, respectively, all of which reached a highly significant level, indicating the existence of common factors between the variables and that they were suitable for factor analysis. Finally, based on the results of the factor analysis of each part of the learning behaviour influencing factors, the learning behaviour, and the learning outcome, deleting the items with similar high loading values in two dimensions, the part of learning behaviour influencing factors contained a total of 20 questions. At last, the scale of factors of influencing learning behaviour were extracted for learning attitude (4 items), learning motivation (7 items), self-efficacy (3 items), and the intention of learning behaviour (6 items). The variation rate explained by the four factors was 63.202%. The learning behaviour section contained 12 questions and three factors were extracted for learning behaviour in class (4 items), completion of assignments (3 items), and extracurricular learning practice (5 items), and the variation rate explained by the three factors was 68.294%. The learning outcome section contained 13 questions and two factors were extracted for capability gains (9 items) and value gains (4 items). The variation rate explained by the two factors was 70.798%. The factor loadings for each item of the question are shown in Table 1.

### 3.3 Model fit test

**Figure 1:**
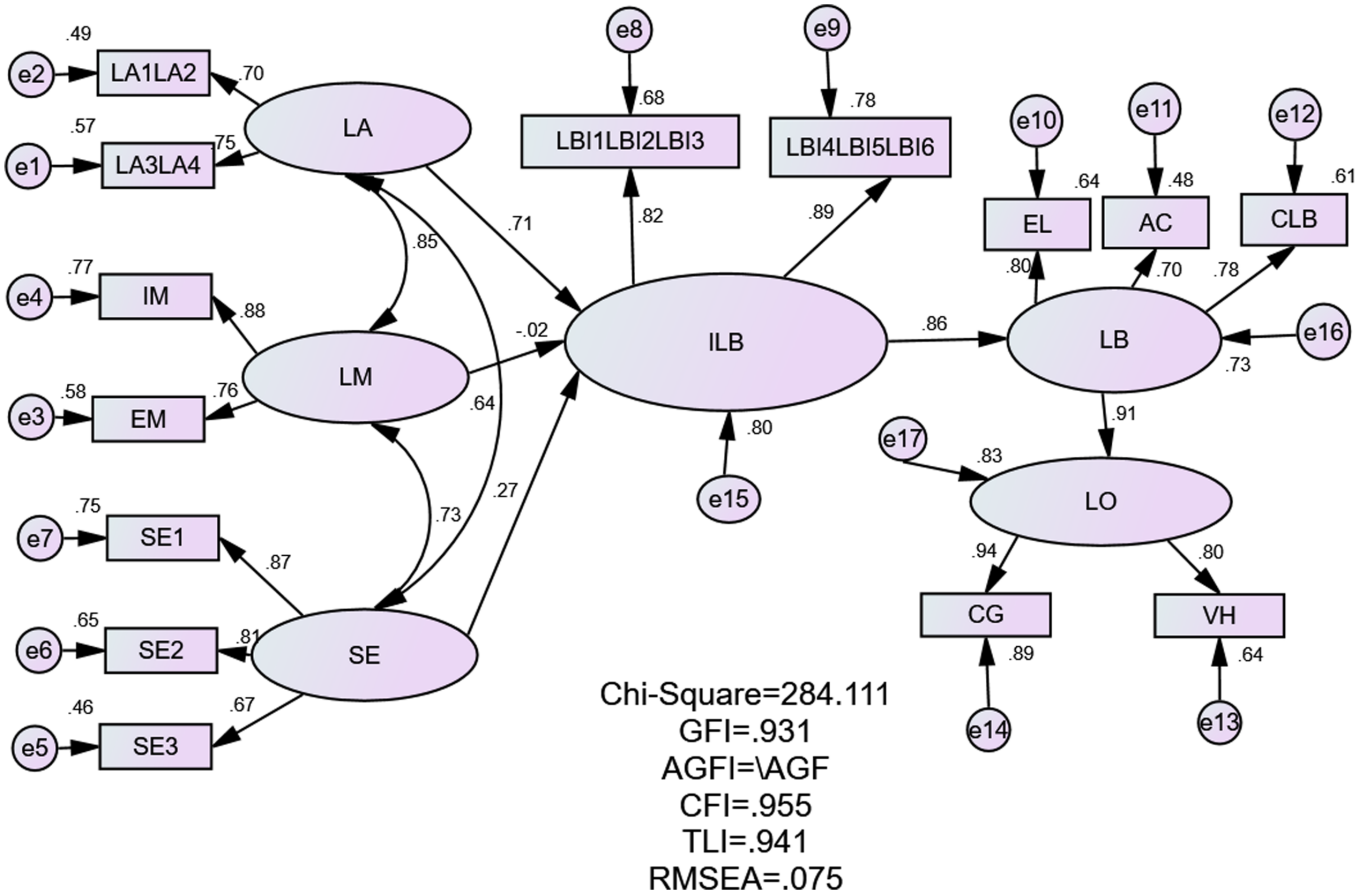
Model fitting diagram.

Based on the exploratory factor analysis, the scale validation factor analysis was performed using AMOS 24.0. The results showed that the chi-square value of the model was 284.111, the degree of freedom was 69, the ratio of chi-square to free degree was 4.118, GFI = 0.931, NFI = 0.942, CFI = 0.955, TLI = 0.941, IFI = 0.956, RMR = 0.023, and RMSEA = 0.075. Each index data achieved an ideal fit, indicating that the research model can be accepted, and the model path analysis is shown in Figure 1.

In order to make the model concise, the item parcelling method was used (Yan and Zhong, 2011). The advantages are to increase commonality and modelling efficiency (Little et al., 2002; Matsunaga, 2008), improve indicator confidence (Coffman and Mac Callum, 2005) and model fit (Hall et al., 1999; Landis et al., 2000; Bandalos, 2002), reduce random errors (Little et al., 2002; Matsunaga, 2008) and non-normality (Bandalos and Finney, 2001), and make the estimates more stable (Little et al., 2002; Matsunaga, 2008) and easier to converge (Little et al., 2002).

### 3.4 Direct effect test

Hypothesis testing is seen based on the results of path analysis, to determine the estimated influence value and the significance value with a significance level of 5%. Hypothesis testing was conducted to determine the effect of LA on the ILB, determine the effect of LM on the ILB, determine the effect of SE on the ILB, determine the effect of ILB on the LB, and determine the effect of LB on the LO. The following Table 2 presents the results of hypotheses testing using path analysis.

**Table 2:** Path analysis test result.

Paths	Estimate	S.E.	C.R.	*p*
LA–ILB	0.713	0.137	4.578	***
LM–ILB	−0.018	0.142	0.067	0.947
SE–LB	0.273	0.079	5.025	***
ILB–LB	0.857	0.108	3.624	***
LB–LO	0.909	0.045	17.801	***

***p < 0.001.

LA affects the IBL with an estimated value of 0.713 and a significance of 0.000***, so the first hypothesis is supported. LM affects the ILB with an estimated value of-0.018 and not have a significant effect, so H2 is not supported. SE affects the ILB with an estimated value of 0.273 and a significance of 0.000***, so the H3 is supported. ILB affects the LB with an estimated value of 0.857 and a significance of 0.000***, so H4 is supported. LB affects the LO with an estimated value of 0.909 and a significance of 0.000***, so H5 is supported.

### 3.5 Indirect effect test

The indirect effect test was used to see the significance of the mediator’s role of IBL in mediating the LA, LM, and SE on the LB. In this test, the bootstrap method is used. Because in most cases this is the strongest and most reliable way to explain the mediating effect of the mediating variable, confidence intervals can be obtained for certain indirect effects (Sucre-Rosales et al., 2020).

Table 3 shows that the indirect of LA on LB through the mediation of IBL with a 95% confidence level of probability ranges from 0.084 to 1.069, and the indirect effect is 0.611***, which lies between these two values. Thus, it can be concluded that the LA has a significant indirect effect on the LB through the mediation of ILB, so H6 is supported. Likewise, the SE on the LB through the mediation of ILB is likely to range from 0.377 to 0.382, and the indirect effect is 0.234***, which lies between these two values, so H8 is supported. But the indirect of LM on LB through the mediation of ILB with a 95% confidence level of probability ranges from-0.447 to 0.268, and the indirect effect is-0.015, which not lies between these two values. H7 is not supported.

**Table 3:** The mediating effect of intention of learning behaviour.

Independent variable	Intermediate variable	Implicit variable	Indirect effects	Bootstrapping BC 95% CI		Toal effects
				Lower limit	Upper limit	
LA	IBL	LB	0.611***	0.377	1.069	0.377
LM	IBL	LB	−0.015	−0.447	0.268	−0.447
SE	IBL	LB	0.234***	0.084	0.382	0.084

***p < 0.001.

## 4 Discussion

Rapid social, technological and cultural changes have also brought dramatic changes in education. Changing educational paradigms have required revising students’ attitudes towards learning, which determine learning abilities and willingness (Güngör, 2023). There is studies have shown that university students’ attitudes predict their success orientations (Entwistle, 2012; Güngör, 2021). Therefore, many countries have adopted strategies to develop students’ attitude to learning as an important objective in their education programmes (Martin et al., 2016). Positive attitudes to learning motivate students’ learning behaviours. The results of this study prove the importance of LA in influencing the ILB. The estimated value of 0.713 with a significance value of 0.000*** is a significant number that shows the significance of LA in ILB. At the same time, LA has a significant effect on LB mediated by the ILB and the estimated value of 0.611 with a significance value of 0.000***. It is clear that ILB is a strong mediating variable, and a good attitude towards learning promotes students’ intention to learn which in turn has an impact on learning behaviour.

However, when ILB was used as a mediating variable, the effect of LM on LB was not significant. Some scholars working on student motivation suggest that LM has direct impact on LB (Martin, 2008; Tokan and Imakulata, 2019). That means that LM has a direct impact on LB, and LM reinforces LB which in turn promote learning outcomes.

SE is a popular construct among researchers interested in student learning and performance. It has been used successfully to explain and predict a variety of cognitive, affective, and behavioural outcomes in diverse academic settings. Evidence has accumulated that unanimously points to the functional advantage of having strong self-efficacy beliefs (Renninger and Hidi, 2019). The results of this study prove the importance of SE in influencing the IBL, and indirectly through the mediation of IBL to LB. The estimated value of 0.273 with a significance value of 0.000*** is a significant number that shows the significance of SE in ILB. Meanwhile, SE has a significant effect on LB mediated by ILB, and the estimated value of 0.234 with a significance value of 0.000***. It suggests that ILB is a strong mediating variable and SE can contribute to students’ intention to learn and thus has an impact on learning behaviour. It is clear that ILB is a strong mediating variable.

In addition, the LB is an important factor in influencing the LO (Swift and Spivack, 1969; McKinney et al., 1975; Kormos and Csizér, 2014). Some studies have shown that learning behaviours such as the pre-study preparation of the course, attendance status, the resource learning situation, resource review, interaction and participation and the completion of learning tasks and enthusiasm all have an impact on LO (Erikson and Erikson, 2018). This is consistent with the results of this study.

## 5 Conclusion

First, LA and SE are significant influencing factors of ILB. LA and SE are an important factor that regulates individual LB and many psychologists regard LA and SE as non-intellectual factors in a central position. Jones (1990) study shown through experimental studies that LA and SE have a moderating effect on LB, as shown by the fact that when students have positive LA and SE, they produce positive LB, which facilitates LO. This study verified these findings through a quantitative analysis. It indicates that when learners have good LA and SE, they tend to be more willing to learn. For graduate students, good learning attitude is extremely important to actively conducting scientific research.

Second, the influence of ILB is extremely significant. Bird suggested that intention is not a simple expectation of a future behaviour but a positive commitment to a future behaviour, mainly because intention reflects the commitment to adopt a particular behaviour in the future and leads individuals to focus their attention on a specific goal and the way to achieve it to realise such behaviour (Bird, 1988). Intention is a strong predictor of actual behaviour. This study prove that ILB plays a mediator role in the relationship between LA, SE, and LB, and is a strong predictor of actual LB of learners. It suggests that the stronger and more pronounced an individual’s intention of learning behaviour, the higher the likelihood of actually engaging in learning. Obviously, ILB is an important driver of learning behaviour.

Third, LB has a significant impact on LO. Research showed that good learning behaviour significantly positively impact learning outcome. Out-of-class learning practices have the most significant positive impact on learning outcomes.

## Data availability statement

The raw data supporting the conclusions of this article will be made available by the authors, without undue reservation.

## Ethics statement

Ethical review and approval were not required for the study on human participants in accordance with the local legislation and institutional requirements. Written informed consent from the patients/participants or patients/participants legal guardian/next of kin was not required to participate in this study in accordance with the national legislation and the institutional requirements.

## Author contributions

XHL: conceptualization, data curation, formal analysis, and resources. ZHJW: methodology and software. XHL and JYX: writing—original draft and writing—review and editing. All authors contributed to the article and approved the submitted version.

## References

[ref1] Ajzen I. (1991). The theory of planned behavior. Organ. Behav. Hum. Decis. Process. 50, 179–211. doi: 10.1016/0749-5978(91)90020-t

[ref2] Anthony G. (2000). Factors influencing first-year students' success in mathematics. Int. J. Math. Educ. Sci. Technol. 31, 3–14. doi: 10.1080/002073900287336

[ref3] Bandalos D. L. (2002). The effects of item parceling on goodness-of-fit and parameter estimate Bias in structural equation modeling. Struct. Equ. Model. Multidiscip. J. 9, 78–102. doi: 10.1207/S15328007SEM0901_5

[ref4] Bandalos D. L. Finney S. J. (2001). Item parceling issues in structural equation modeling, 269–296. New Jersey: Lawrence Erlbaum Associates.

[ref5] Bandura A. Schunk D. H. (1981). Cultivating competence, self-efficacy, and intrinsic interest through proximal self-motivation. J. Pers. Soc. Psychol. 41, 586–598. doi: 10.1037//0022-3514.41.3.586

[ref6] Bird B. (1988). Implementing entrepreneurial ideas: the case for intention. Acad. Manag. Rev. 13, 442–453. doi: 10.2307/258091

[ref7] Bosch E. Seifried E. Spinath B. (2021). What successful students do: evidence-based learning activities matter for students' performance in higher education beyond prior knowledge, motivation, and prior achievement. Learn. Individ. Differ. 91:102056. doi: 10.1016/j.lindif.2021.102056

[ref8] Carini R. M. Kuh G. D. Klein S. P. (2006). Student engagement and student learning: testing the linkages*. Res. High. Educ. 47, 1–32. doi: 10.1007/s11162-005-8150-9

[ref9] Chao J. L. Mcdermott P. A. Watkins M. W. Drogalis A. R. Worrell F. C. Hall T. E. (2018). The learning behaviors scale: national standardization in Trinidad and Tobago. Int. J. Sch. Educ. Psychol. 6, 35–49. doi: 10.1080/21683603.2016.1261055

[ref10] Coffman D. L. Mac Callum R. C. (2005). Using parcels to convert path analysis models into latent variable models. Multivar. Behav. Res. 40, 235–259. doi: 10.1207/s15327906mbr4002_4, PMID: 26760108

[ref11] David T. Mohamad R. R. (2006). Diagnosing students’ difficulties in learning mathematics. Int. J. Math. Educ. Sci. Technol. 24, 209–222. doi: 10.1080/0020739930240206

[ref12] Deqing T. (2001). Theory and research on learning attitudes. 119–124. Guangdong: Guangdong People's Publishing House.

[ref13] Durbrow E. H. Schaefer B. A. Jimerson S. R. (2000). Learning behaviours, attention and anxiety in Caribbean children: beyond the usual suspects in explaining academic performance. Sch. Psychol. Int. 21, 242–251. doi: 10.1177/0143034300213002

[ref14] Dzewaltowski D. A. Noble J. M. Shaw J. M. (1990). Physical activity participation: social cognitive theory versus the theories of reasoned action and planned behavior. J. Sport Exerc. Psychol. 12, 388–405. doi: 10.1123/jsep.12.4.388, PMID: 28796958

[ref15] Eccles J. S. Wigfield A. Schiefele U. (1998). “Motivation to succeed” in Handbook of child psychology: Social, emotional, and personality development. eds. Damon W. Eisenberg N. (US: John Wiley & Sons, Inc), 1017–1095.

[ref16] Elliot A. J. (1999). Approach and avoidance motivation and achievement goals. Educ. Psychol. 34, 169–189. doi: 10.1207/s15326985ep3403_3

[ref17] Elliot A. J. McGregor H. A. (2001). A 2 × 2 achievement goal framework. J. Pers. Soc. Psychol. 80, 501–519. doi: 10.1037/0022-3514.80.3.501 11300582

[ref18] Entwistle N. J. (2012). “Approaches to learning and studying” in Encyclopedia of the sciences of learning. ed. Seel N. M. (Boston, MA: Springer), 288–291.

[ref19] Erikson M. G. Erikson M. (2018). Learning outcomes and critical thinking – good intentions in conflict. Stud. High. Educ. 44, 2293–2303. doi: 10.1080/03075079.2018.1486813

[ref20] Escalón X. D. Greenfield D. (2009). Learning behaviors mediating the effects of behavior problems on academic outcomes. NHSA Dialog. 12, 1–17. doi: 10.1080/15240750802590768

[ref21] Froehlich D. E. Raemdonck I. Beausaert S. (2023). Resources to increase older Workers' motivation and intention to learn. Vocat. Learn. 16, 47–71. doi: 10.1007/s12186-022-09304-9

[ref22] Gatch C. L. Kendzierski D. (1990). Predicting exercise intentions: the theory of planned behavior. Res. Q. Exerc. Sport 61, 100–102. doi: 10.1080/02701367.1990.10607485 2091158

[ref23] Geitz G. Brinke D. J. Kirschner P. A. (2016). Changing learning behaviour: self-efficacy and goal orientation in PBL groups in higher education. Int. J. Educ. Res. 75, 146–158. doi: 10.1016/j.ijer.2015.11.001

[ref24] Güngör C. (2021). The relationship between attitudes towards learning and success orientation in undergraduate students. Int. Online J. Educ. Teach. (IOJET) 8, 1774–1796.

[ref25] Güngör C. (2023). Socially and educationally distanced: examining the views of university students. SAGE Open 13:215824402311736. doi: 10.1177/21582440231173664

[ref26] Hall R. J. Snell A. F. Foust M. S. (1999). Item parceling strategies in SEM: investigating the subtle effects of Unmodeled secondary constructs. Organ. Res. Methods 2, 233–256. doi: 10.1177/109442819923002

[ref27] Harter S. (1981). A new self-report scale of intrinsic versus extrinsic orientation in the classroom: motivational and informational components. Dev. Psychol. 17, 300–312. doi: 10.1037/0012-1649.17.3.300

[ref28] Hsieh H. Hsieh H. (2019). Undergraduates’ out-of-class learning: exploring EFL students’ autonomous learning behaviors and their usage of resources. Educ. Sci. 9, 12–17. doi: 10.3390/educsci9030159

[ref29] Huang X. (2022). Relationship between learning behavior and learning effect based on big data. Lecture notes on data engineering and communications technologies. 102. Singapore: Springer.

[ref30] Hwang G. Wang S. Lai C. (2021). Effects of a social regulation-based online learning framework on students' learning achievements and behaviors in mathematics. Comput. Educ. 160:104031. doi: 10.1016/j.compedu.2020.104031

[ref31] Jaedun A. Nurtanto M. Mutohhari F. Majid N. W. A. Kurdi M. S. (2022). Mediating role of teachers’ self-efficacy and psychological capital in determining success during learning transition periods in vocational education. J. Educ. E-Learn. Res. 9, 207–215. doi: 10.20448/jeelr.v9i3.4193

[ref32] Jones E. E. (1990). Interpersonal perception. pp: 313. New York: W.H. Freeman.

[ref33] Jovanović J. Saqr M. Joksimovíc S. Gašević D. (2021). Students matter the most in learning analytics: the effects of internal and instructional conditions in predicting academic success. Comput. Educ. 172:104251. doi: 10.1016/j.compedu.2021.104251

[ref34] Keengwe J. Bhargava M. (2014). Mobile learning and integration of mobile technologies in education. Educ. Inf. Technol. 19, 737–746. doi: 10.1007/s10639-013-9250-3

[ref35] Kholifah N. Kurdi M. S. Nurtanto M. Mutohhari F. Fawaid M. Subramaniam T. S. (2023). The role of teacher self-efficacy on the instructional quality in 21st century: a study on vocational teachers, Indonesia. Int. J. Evaluation and Res. Educ. (IJERE) 12, 998–1006. doi: 10.11591/IJERE.V12I2.23949

[ref36] Klug J. Bruder S. Kelava A. Spiel C. Schmitz B. (2013). Diagnostic competence of teachers: a process model that accounts for diagnosing learning behavior tested by means of a case scenario. Teach. Teach. Educ. 30, 38–46. doi: 10.1016/j.tate.2012.10.004

[ref37] Kormos J. Csizér K. (2014). The interaction of motivation, self-regulatory strategies, and autonomous learning behavior in different learner groups. TESOL Q. 48, 275–299. doi: 10.1002/tesq.129

[ref38] Landis R. S. Beal D. J. Tesluk P. E. (2000). A comparison of approaches to forming composite measures in structural equation models. Organ. Res. Methods 3, 186–207. doi: 10.1177/109442810032003

[ref39] Linnenbrink E. A. Pintrich P. R. (2002). Motivation as an enabler for academic success. Sch. Psychol. Rev. 31, 313–327. doi: 10.1080/02796015.2002.12086158

[ref40] Little T. D. Cunningham W. A. Shahar G. Widaman K. F. (2002). To parcel or not to parcel: exploring the question, weighing the merits. Struct. Equ. Model. Multidiscip. J. 9, 151–173. doi: 10.1207/S15328007sem0902_1

[ref41] Locke E. A. (1997). Self-efficacy: the exercise of control. Pers. Psychol. 50, 801–804.

[ref42] Loewenstein G. F. Weber E. U. Hsee C. K. Welch N. (2001). Risk as feelings. Psychol. Bull. 127, 267–286. doi: 10.1037/0033-2909.127.2.267 11316014

[ref43] Martin A. J. (2008). Enhancing student motivation and engagement: the effects of a multidimensional intervention. Contemp. Educ. Psychol. 33, 239–269. doi: 10.1016/j.cedpsych.2006.11.003

[ref44] Martin M. O. Mullis I. V. S. Hooper M. (Eds.). (2016). Methods and procedures in TIMSS advanced 2015. Retrieved from Boston College, TIMSS & PIRLS international study center website: http://timss.bc.edu/publications/timss/2015-a-methods.html

[ref45] Matsunaga M. (2008). Item parceling in structural equation modeling: a primer. Commun. Methods Meas. 2, 260–293. doi: 10.1080/19312450802458935

[ref46] Mcdermott P. A. (1999). National scales of differential learning behaviors among American children and adolescents. Sch. Psychol. Rev. 28, 280–291. doi: 10.1080/02796015.1999.12085965

[ref47] Mcdermott P. A. Leigh N. M. Perry M. A. (2002). Development and validation of the preschool learning behaviors scale. Psychol. Sch. 39, 353–365. doi: 10.1002/pits.10036

[ref48] Mcdermott P. A. Rikoon S. Waterman C. Fantuzzo J. W. (2012). The preschool learning behaviors scale: dimensionality and external validity in head start. Sch. Psychol. Rev. 41, 66–81. doi: 10.1080/02796015.2012.12087376

[ref49] Mckinney J. D. Mason J. Perkerson K. Clifford M. (1975). Relationship between classroom behavior and academic achievement. J. Educ. Psychol. 67, 198–203. doi: 10.1037/h0077012

[ref9001] NSSE (2019). Available at: https://und.edu/analytics-and-planning/_files/docs/_files/nsse-2019-survey-nstrument.pdf

[ref50] OECD (2004). “Student learning: attitudes, engagement and strategies” in Learning for Tomorrow's world: First results from PISA 2003 (Paris: OECD Publishing)

[ref51] Pawlak M. Csizér K. Soto A. (2020). Interrelationships of motivation, self-efficacy and self-regulatory strategy use: an investigation into study abroad experiences. System 93:102300. doi: 10.1016/j.system.2020.102300

[ref52] Pintrich P. R. Smith D. A. F. Garcia T. Mckeachie W. J. (1993). Reliability and predictive validity of the motivated strategies for learning questionnaire (Mslq). Educ. Psychol. Meas. 53, 801–813. doi: 10.1177/0013164493053003024

[ref53] Rahman R. A. Mason J. Yusof Y. M. (2012). Factors affecting students’ change of learning behaviour. Procedia Soc. Behav. Sci. 56, 213–222. doi: 10.1016/j.sbspro.2012.09.648

[ref54] Renninger K. A. Hidi S. E. (Eds.). (2019). The Cambridge handbook of motivation and learning. 63–86. Cambridge: Cambridge University Press.

[ref55] Reynolds W. M. (1979). Development and validation of a scale to measure learning-related classroom behaviors. Educ. Psychol. Meas. 39, 1011–1018. doi: 10.1177/001316447903900441

[ref56] Rikoon S. Mcdermott P. A. Fantuzzo J. W. (2012). Approaches to learning among head start alumni: structure and validity of the learning behaviors scale. Sch. Psychol. Rev. 41, 272–294. doi: 10.1080/02796015.2012.12087509

[ref57] Sedrakyan G. Malmberg J. Verbert K. Järvelä S. Kirschner P. A. (2020). Linking learning behavior analytics and learning science concepts: designing a learning analytics dashboard for feedback to support learning regulation. Comput. Hum. Behav. 107:105512. doi: 10.1016/j.chb.2018.05.004

[ref58] Sichler R. (2014). “Motivation, overview” in Encyclopedia of critical psychology. ed. Teo T. (New York: Springer), 1204–1209.

[ref59] Singh M. James P. S. Paul H. Bolar K. (2022). Impact of cognitive-behavioral motivation on student engagement. Heliyon 8:e09843. doi: 10.1016/j.heliyon.2022.e09843, PMID: 35815149 PMC9264003

[ref60] Sondeng S. Nurwati Sukotjo E. Maharani S. (2020). Model of organizational learning culture on knowledge sharing behavior. Int. J. Biometrics 15, 184–191. doi: 10.5539/ijbm.v15n11p184

[ref61] Spivack G. Swift M. S. (1966). The Devereux elementary school behavior rating scales: a study of the nature and Organization of Achievement Related Disturbed Classroom Behavior. J. Spec. Educ. 1, 71–90. doi: 10.1177/002246696600100109

[ref62] Sucre-Rosales E. Fernández-Terán R. J. Carvajal D. Echevarria L. Hernández F. E. (2020). Experience-based learning approach to chemical kinetics: learning from the COVID-19 pandemic. J. Chem. Educ. 97, 2598–2605. doi: 10.1021/acs.jchemed.0c00698

[ref63] Swift M. S. Spivack G. (1969). Clarifying the relationship between academic success and overt classroom behavior. Except. Child. 36, 99–104. doi: 10.1177/001440296903600205, PMID: 5351705

[ref64] Tokan M. K. Imakulata M. M. (2019). The effect of motivation and learning behaviour on student achievement. S. Afr. J. Educ. 39, 1–8. doi: 10.15700/saje.v39n1a1510

[ref65] Valaitis R. K. Sword W. Jones B. Hodges A. (2005). Problem-based learning online: perceptions of health science students. Adv. Health Sci. Educ. 10, 231–252. doi: 10.1007/s10459-005-6705-3 16193403

[ref66] Van Uum M. S. Pepin B. (2022). Students’ self-reported learning gains in higher engineering education. Eur. J. Eng. Educ. 48, 42–58. doi: 10.1080/03043797.2022.2046708

[ref67] Yan W. Zhong L. W. (2011). Item parceling strategies in structural equation modeling. Adv. Psychol. Sci. 19, 1859–1867.

[ref68] Yen C.-J. Konold T. R. McDermott P. A. (2004). Does learning behavior augment cognitive ability as an indicator of academic achievement? J. Sch. Psychol. 42, 157–169. doi: 10.1016/j.jsp.2003.12.001

[ref69] Zimmerman B. (2000). Self-efficacy: an essential motive to learn. Contemp. Educ. Psychol. 25, 82–91. doi: 10.1006/ceps.1999.1016 10620383

